# Absolute configuration of xerophenone A

**DOI:** 10.1107/S1600536812015267

**Published:** 2012-04-21

**Authors:** Hoong-Kun Fun, Cholpisut Tantapakul, Surat Laphookhieo, Nawong Boonnak, Suchada Chantrapromma

**Affiliations:** a X-ray Crystallography Unit, School of Physics, Universiti Sains Malaysia, 11800 USM, Penang, Malaysia; b Natural Products Research Laboratory, School of Science, Mae Fah Luang University, Tasud, Muang Chiang Rai 57100, Thailand; c Crystal Materials Research Unit, Department of Chemistry, Faculty of Science, Prince of Songkla University, Hat-Yai, Songkhla 90112, Thailand

## Abstract

The title compound, C_33_H_42_O_5_, known as xerophenone A {systematic name: (1*R*,3*R*,4*R*,6*S*,8*E*,10*R*)-10-hy­droxy-8-[hy­droxy(phen­yl)methyl­ene]-4-methyl-1,6-bis­(3-methyl­but-2-en-1-yl)-3-(3-methyl­but-3-en-1-yl)-11-oxatricyclo­[4.3.1.1^4,10^]undecane-7,9-dione} is a naturally occurring rearranged benzophenone compound which was isolated from the twigs of *Garcinia propinqua*. The absolute configuration was determined by refining the Flack parameter to 0.18 (16). The absolute configurations at positions 1, 3, 4, 6 and 10 of the xerophenone A are *R*, *R*, *R*, *S* and *R*. In the mol­ecule, the cyclo­hexane-1,3-dione, tetra­hydro-2*H*-pyran and tetra­hydro­furan rings adopt twisted boat, standard chair and envelope conformations, respectively. The 3-methyl­but-3-en-1-yl substituent is disordered over two sets of sites in a 0.771 (11):0.229 (11) ratio. An intra­molecular O—H⋯O hydrogen bond generates an *S*(6) ring motif. In the crystal, mol­ecules are linked by O—H⋯O and weak C—H⋯O inter­actions into a chain along the *a* axis. A very weak C—H⋯π inter­action and C⋯O short contact [2.989 (2) Å] are also present.

## Related literature

For bond-length data, see: Allen *et al.* (1987 ▶). For ring conformations, see: Cremer & Pople (1975 ▶). For hydrogen-bond motifs, see: Bernstein *et al.* (1995 ▶). For background to plants in the Clusiaceae family, bioactive metabolites and their biological and pharmacological activities, see: Castardo *et al.* (2008 ▶); Henry & Jacobs (1995 ▶); Nguyen *et al.* (2011 ▶); Phongpaichit *et al.* (1994 ▶); Suksamrarn *et al.* (2006 ▶); Thoison *et al.* (2005 ▶); Xu *et al.* (2010 ▶); Yu *et al.* (2007 ▶). For the stability of the temperature controller used in the data collection, see: Cosier & Glazer (1986 ▶).
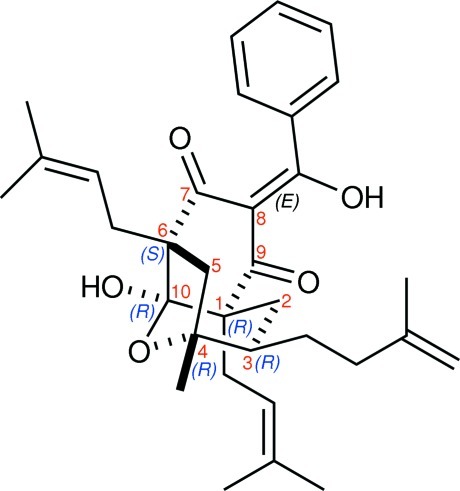


## Experimental

### 

#### Crystal data

C_33_H_42_O_5_
*M* *_r_* = 518.67
Monoclinic, 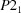

*a* = 6.1984 (2) Å
*b* = 17.0998 (4) Å
*c* = 13.7007 (3) Å
β = 91.036 (1)°
*V* = 1451.92 (7) Å^3^
*Z* = 2
Cu *K*α radiation
μ = 0.62 mm^−1^
*T* = 100 K
0.31 × 0.13 × 0.09 mm

#### Data collection

Bruker APEX Duo CCD area detector diffractometer
Absorption correction: multi-scan (*SADABS*; Bruker, 2009 ▶) *T*_min_ = 0.830, *T*_max_ = 0.944
11622 measured reflections
4746 independent reflections
4661 reflections with *I* > 2σ(*I*)
*R*_int_ = 0.023

#### Refinement

*R*[*F* ^2^ > 2σ(*F* ^2^)] = 0.037
*wR*(*F* ^2^) = 0.099
*S* = 1.07
4746 reflections
369 parameters
7 restraints
H-atom parameters constrained
Δρ_max_ = 0.24 e Å^−3^
Δρ_min_ = −0.20 e Å^−3^
Absolute structure: Flack (1983 ▶), 2079 Friedel pairs
Flack parameter: 0.18 (16)

### 

Data collection: *APEX2* (Bruker, 2009 ▶); cell refinement: *SAINT* (Bruker, 2009 ▶); data reduction: *SAINT*; program(s) used to solve structure: *SHELXTL* (Sheldrick, 2008 ▶); program(s) used to refine structure: *SHELXTL*; molecular graphics: *SHELXTL*; software used to prepare material for publication: *SHELXTL* and *PLATON* (Spek, 2009 ▶).

## Supplementary Material

Crystal structure: contains datablock(s) global, I. DOI: 10.1107/S1600536812015267/is5112sup1.cif

Structure factors: contains datablock(s) I. DOI: 10.1107/S1600536812015267/is5112Isup2.hkl

Supplementary material file. DOI: 10.1107/S1600536812015267/is5112Isup3.cml

Additional supplementary materials:  crystallographic information; 3D view; checkCIF report

## Figures and Tables

**Table 1:** Hydrogen-bond geometry (Å, °) *Cg*4 is the centroid of the C28–C33 ring.

*D*—H⋯*A*	*D*—H	H⋯*A*	*D*⋯*A*	*D*—H⋯*A*
O1—H1*O*1⋯O2	0.82	1.70	2.4446 (19)	151
O4—H1*O*4⋯O3^i^	0.82	1.97	2.7499 (18)	159
C21—H21*B*⋯O3^i^	0.97	2.48	3.283 (2)	139
C25—H25*B*⋯*Cg*4^ii^	0.96	2.99	3.786 (3)	142

Symmetry codes: (i) 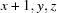; (ii) 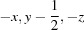.

**Fig. 1.**
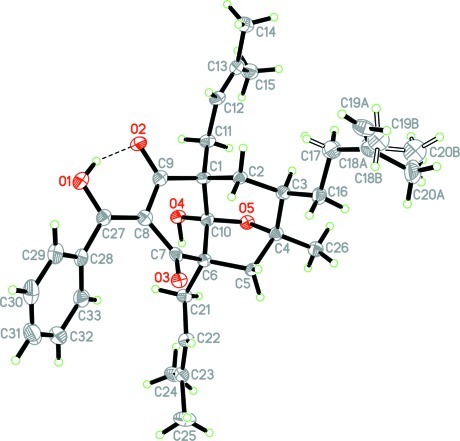
The molecular structure of the title compound, showing 50% probability displacement ellipsoids and the atom-numbering scheme. Open bond is a minor component. The intramolecular O—H···O hydrogen bond is shown as a dashed line.

**Fig. 2.**
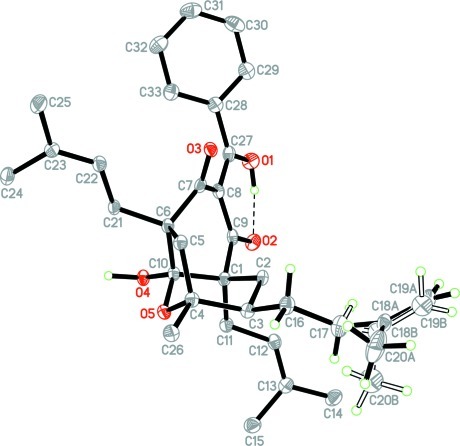
The molecular structure of the title compound, showing 40% probability displacement ellipsoids and the atom-numbering scheme. The intramolecular O—H···O hydrogen bond was shown as a dashed line. Only H atoms of the hydroxy groups and the disordered side chain substituent are shown for clarity.

**Fig. 3.**
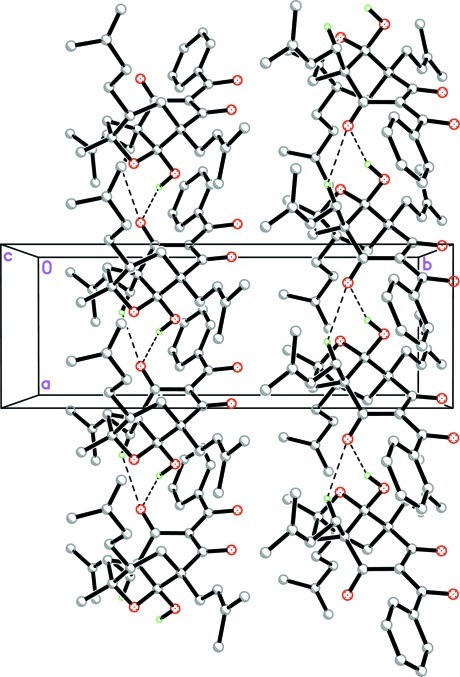
The crystal packing diagram of the major component of the title compound, viewed along the c axis. Only H atoms involving in the hydrogen bonds (dashed lines) are shown.

## supplementary crystallographic information

### Comment

*Garcinia* belongs to the Clusiaceae family which is well recognized to produce a variety of biological active metabolites including xanthones (Phongpaichit *et al.*, 1994), flavanones (Castardo *et al.*, 2008), terpenoids (Nguyen *et al.*, 2011) and benzophenones (Thoison *et al.*, 2005; Xu *et al.*, 2010). Several of these compounds possess interesting biological and pharmacological activities, such as antimicrobial (Xu *et al.*, 2010), antidepressant and anti-HIV (Xu *et al.*, 2010), antioxidant (Yu *et al.*, 2007) and also cytotoxic (Suksamrarn *et al.*, 2006) activities. During the course of our study of bioactive compounds from medicinal plants, the title compound (I), known as xerophenone A (Henry & Jacobs, 1995) was isolated from the twigs of *G. propinqua* which were collected from Chiang Rai province in the northern part of Thailand. Herein the crystal structure and absolute configuration of (I) was reported.

The title compound (I), C_33_H_42_O_5_, is a rearranged benzophenone and an isoprenylated derivative of 11-oxatricyclo[4.3.1.1^4,10^]undecane-7,9-dione. Figure 1 shows that (I) exists in an *E* configuration with respect to the C8═C27 double bond [1.402 (3) Å] with a C9–C8–C27–C28 torsion angle 177.48 (17)°. In order to view more clearly and gain more information of the ring conformations and the orientations of the rings and how each substituent is in respect to the bound rings, Figure 2 is also shown. It can be clearly seen that the cyclohexane-1,3-dione ring is in a twisted boat with the puckering parameters Q = 0.616 (2) Å, θ = 101.86 (19)° and φ = 157.84 (19)° (Cremer & Pople, 1975), the tetrahydro-2*H*-pyran ring (C1–C4/C10/O5) is in a standard chair conformation and the tetrahydrofuran ring (C4–C6/C10/O5) is in an envelope conformation with the puckering atom O5 of 0.283 (1) Å, and puckering parameter Q = 0.4223 (18) Å and φ = 1.0 (3)°. The two 3-methyl-2-buten-1-yl substituents (C11–C15 and C21–C15) are attached to the cyclohexane-1,3-dione moiety at atoms C1 and C6 with the torsion angles C1—C11—C12–C13 = 153.3 (2)° and C6–C21–C22–C23 = -161.87 (19)°, indicating a (+)-anti-periplanar and (-)-anti-periplanar conformations, respectively. The 3-methyl-3-buten-1-yl substituent (C16–C20) is disordered over two positions in a 0.771 (11): 0.229 (11) ratio, and attached to the tetrahydro-2*H*-pyran ring at atom C3 with the torsion angles C3–C16–C17–C18 = 179.6 (3)° for the major component (*A*) and -168.3 (8)° for the minor component (*B*) (Fig. 1). An intramolecular O1—H1O1···O2 hydrogen bond (Table 1) generates an S(6) ring motif (Bernstein *et al.*, 1995). The bond distances in (I) are within normal ranges (Allen *et al.*, 1987). The absolute configuration at atoms C1, C3, C4, C6 and C10 or at positions 1, 3, 4, 6 and 10 of the xerophenone A are *R*,*R*,*R*,*S*,*R* configurations.

In the crystal (Fig. 3), molecules are linked by O—H···O hydrogen bonds and weak C—H···O interactions (Table 1) forming an *R*^2^_1_(7) motif which connected the molecules into a chain along the *a* axis. A C—H···π interaction (Table 1) and a C27···O4^i^ short contact [2.989 (2) Å] are observed.

### Experimental

Twigs of *G. propinqua* (1.90 Kg) were successively extracted with acetone over the period of 3 days at room temperature to provide the crude acetone extract (183.72g) which was subjected to quick column chromatography over silica gel and eluted by gradients of EtOAc-hexanes (100% hexanes to 100% EtOAc) to yield fourteen fractions (A-N). Fraction B (268.0 mg) was subjected to sephadex LH-20 using CH_3_OH as eluent to afford compound (I) (21.8 mg) as white solid. Colorless plate-shaped single crystals of the title compound suitable for *x*-ray structure determination were recrystallized from CH_2_Cl_2_:acetone (1:1 v/v) by the slow evaporation of the solvent at room temperature after several days, m.p. 451.0-452.4 K [[α]^D^_29_ -21.2 (*c* 0.400, CH_3_OH)].

### Refinement

H atoms were placed in calculated positions with d(O—H) = 0.82 Å, and d(C—H) = 0.93 Å for aromatic and CH, 0.97 Å for CH_2_ and 0.96 Å for CH_3_ atoms. The *U*_iso_(H) values were constrained to be 1.5*U*_eq_ of the carrier atom for methyl H atoms and 1.2*U*_eq_ for the remaining H atoms. A rotating group model was used for the methyl groups. The 3-methyl-3-buten-1-yl substituent was found to be disordered over two sites in a 0.771 (11): 0.229 (11) occupancy ratio. In the final refinement, distance restraints were used for the disordered components. 2079 Friedel pairs were used to determine the absolute configuration.

### Crystal data

**Table:**

C_33_H_42_O_5_	*F*(000) = 560
*M**_r_* = 518.67	*D*_x_ = 1.186 Mg m^−^^3^
Monoclinic, *P*2_1_	Melting point = 451.0–452.4 K
Hall symbol: P 2yb	Cu *K*α radiation, λ = 1.54178 Å
*a* = 6.1984 (2) Å	Cell parameters from 4746 reflections
*b* = 17.0998 (4) Å	θ = 3.2–67.5°
*c* = 13.7007 (3) Å	µ = 0.62 mm^−^^1^
β = 91.036 (1)°	*T* = 100 K
*V* = 1451.92 (7) Å^3^	Plate, colorless
*Z* = 2	0.31 × 0.13 × 0.09 mm

### Data collection

**Table:**

Bruker APEX Duo CCD area detector diffractometer	4746 independent reflections
Radiation source: sealed tube	4661 reflections with *I* > 2σ(*I*)
Graphite monochromator	*R*_int_ = 0.023
φ and ω scans	θ_max_ = 67.5°, θ_min_ = 3.2°
Absorption correction: multi-scan (*SADABS*; Bruker, 2009)	*h* = −6→7
*T*_min_ = 0.830, *T*_max_ = 0.944	*k* = −19→20
11622 measured reflections	*l* = −16→16

### Refinement

**Table:**

Refinement on *F*^2^	Secondary atom site location: difference Fourier map
Least-squares matrix: full	Hydrogen site location: inferred from neighbouring sites
*R*[*F*^2^ > 2σ(*F*^2^)] = 0.037	H-atom parameters constrained
*wR*(*F*^2^) = 0.099	*w* = 1/[σ^2^(*F*_o_^2^) + (0.0526*P*)^2^ + 0.4822*P*] where *P* = (*F*_o_^2^ + 2*F*_c_^2^)/3
*S* = 1.07	(Δ/σ)_max_ = 0.001
4746 reflections	Δρ_max_ = 0.24 e Å^−^^3^
369 parameters	Δρ_min_ = −0.20 e Å^−^^3^
7 restraints	Absolute structure: Flack (1983), 2079 Friedel pairs
Primary atom site location: structure-invariant direct methods	Flack parameter: 0.18 (16)

### Special details

**Table:**

Experimental. The crystal was placed in the cold stream of an Oxford Cryosystems Cobra open-flow nitrogen cryostat (Cosier & Glazer, 1986) operating at 100.0 (1) K.
Geometry. All e.s.d.'s (except the e.s.d. in the dihedral angle between two l.s. planes) are estimated using the full covariance matrix. The cell e.s.d.'s are taken into account individually in the estimation of e.s.d.'s in distances, angles and torsion angles; correlations between e.s.d.'s in cell parameters are only used when they are defined by crystal symmetry. An approximate (isotropic) treatment of cell e.s.d.'s is used for estimating e.s.d.'s involving l.s. planes.
Refinement. Refinement of *F*^2^ against ALL reflections. The weighted *R*-factor *wR* and goodness of fit *S* are based on *F*^2^, conventional *R*-factors *R* are based on *F*, with *F* set to zero for negative *F*^2^. The threshold expression of *F*^2^ > σ(*F*^2^) is used only for calculating *R*-factors(gt) *etc*. and is not relevant to the choice of reflections for refinement. *R*-factors based on *F*^2^ are statistically about twice as large as those based on *F*, and *R*- factors based on ALL data will be even larger.

### Fractional atomic coordinates and isotropic or equivalent isotropic displacement parameters (Å^2^)

**Table:**

	*x*	*y*	*z*	*U*_iso_*/*U*_eq_	Occ. (<1)
O1	−0.2134 (2)	0.52353 (8)	0.24048 (11)	0.0296 (3)	
H1O1	−0.1378	0.5351	0.2881	0.084 (13)*	
O2	0.0272 (2)	0.50870 (8)	0.38262 (10)	0.0262 (3)	
O3	−0.2215 (2)	0.27900 (8)	0.24870 (9)	0.0200 (3)	
O4	0.4496 (2)	0.36790 (8)	0.32243 (9)	0.0208 (3)	
H1O4	0.5236	0.3382	0.2901	0.031*	
O5	0.4021 (2)	0.26998 (8)	0.43398 (9)	0.0198 (3)	
C1	0.1716 (3)	0.38478 (11)	0.43458 (13)	0.0191 (4)	
C2	0.0072 (3)	0.33858 (11)	0.49672 (13)	0.0191 (4)	
H2A	−0.0471	0.3725	0.5474	0.023*	
H2B	−0.1140	0.3231	0.4553	0.023*	
C3	0.1071 (3)	0.26536 (11)	0.54423 (13)	0.0212 (4)	
H3A	0.2122	0.2833	0.5936	0.025*	
C4	0.2327 (3)	0.21824 (11)	0.46743 (14)	0.0205 (4)	
C5	0.1006 (3)	0.20180 (11)	0.37297 (13)	0.0193 (4)	
H5A	−0.0517	0.1970	0.3867	0.023*	
H5B	0.1491	0.1541	0.3421	0.023*	
C6	0.1428 (3)	0.27366 (11)	0.30699 (13)	0.0184 (4)	
C7	−0.0628 (3)	0.31594 (11)	0.27620 (12)	0.0192 (4)	
C8	−0.0625 (3)	0.40194 (12)	0.28183 (13)	0.0210 (4)	
C9	0.0452 (3)	0.43657 (12)	0.36486 (14)	0.0212 (4)	
C10	0.2965 (3)	0.32512 (11)	0.37240 (13)	0.0189 (4)	
C11	0.3343 (3)	0.43234 (12)	0.49784 (14)	0.0229 (4)	
H11A	0.4442	0.3969	0.5228	0.027*	
H11B	0.4050	0.4699	0.4561	0.027*	
C12	0.2397 (3)	0.47577 (11)	0.58299 (14)	0.0236 (4)	
H12A	0.0954	0.4903	0.5773	0.028*	
C13	0.3434 (3)	0.49531 (12)	0.66554 (14)	0.0251 (4)	
C14	0.2342 (4)	0.53943 (14)	0.74493 (15)	0.0340 (5)	
H14A	0.0824	0.5428	0.7303	0.051*	
H14B	0.2938	0.5912	0.7495	0.051*	
H14C	0.2563	0.5127	0.8059	0.051*	
C15	0.5786 (4)	0.47874 (14)	0.68566 (16)	0.0320 (5)	
H15A	0.6407	0.4549	0.6293	0.048*	
H15B	0.5926	0.4439	0.7404	0.048*	
H15C	0.6525	0.5268	0.7002	0.048*	
C16	−0.0659 (3)	0.21819 (12)	0.59759 (14)	0.0262 (4)	
H16A	−0.0041	0.1691	0.6200	0.031*	
H16B	−0.1835	0.2062	0.5524	0.031*	
C17	−0.1550 (4)	0.26321 (14)	0.68556 (17)	0.0355 (5)	
H17A	−0.0353	0.2846	0.7222	0.043*	
H17B	−0.2138	0.3120	0.6628	0.043*	0.771 (12)
H17C	−0.2384	0.3065	0.6610	0.043*	0.229 (12)
C18A	−0.3256 (9)	0.2211 (3)	0.7408 (5)	0.0243 (12)	0.771 (12)
C19A	−0.5160 (5)	0.2548 (3)	0.7573 (2)	0.0322 (9)	0.771 (12)
H19A	−0.6209	0.2279	0.7916	0.038 (9)*	0.771 (12)
H19B	−0.5436	0.3051	0.7345	0.051 (11)*	0.771 (12)
C20A	−0.2778 (9)	0.1407 (2)	0.7766 (3)	0.0546 (17)	0.771 (12)
H20A	−0.3932	0.1230	0.8168	0.082*	0.771 (12)
H20B	−0.1454	0.1410	0.8141	0.082*	0.771 (12)
H20C	−0.2637	0.1060	0.7219	0.082*	0.771 (12)
C18B	−0.289 (2)	0.2169 (14)	0.7528 (18)	0.036 (7)*	0.229 (12)
C19B	−0.500 (2)	0.2237 (11)	0.7641 (10)	0.035 (4)*	0.229 (12)
H19C	−0.5694	0.1940	0.8110	0.043*	0.229 (12)
H19D	−0.5794	0.2582	0.7250	0.043*	0.229 (12)
C20B	−0.162 (2)	0.1622 (9)	0.8146 (11)	0.052 (4)*	0.229 (12)
H20D	−0.2429	0.1489	0.8714	0.078*	0.229 (12)
H20E	−0.0289	0.1866	0.8343	0.078*	0.229 (12)
H20F	−0.1321	0.1156	0.7781	0.078*	0.229 (12)
C21	0.2596 (3)	0.25027 (11)	0.21211 (14)	0.0206 (4)	
H21A	0.2787	0.2968	0.1727	0.025*	
H21B	0.4020	0.2307	0.2296	0.025*	
C22	0.1440 (3)	0.18912 (12)	0.15065 (14)	0.0223 (4)	
H22A	−0.0018	0.1809	0.1619	0.027*	
C23	0.2358 (3)	0.14645 (12)	0.08194 (13)	0.0230 (4)	
C24	0.4683 (3)	0.15337 (14)	0.05475 (15)	0.0299 (5)	
H24A	0.5413	0.1878	0.0997	0.045*	
H24B	0.4776	0.1740	−0.0102	0.045*	
H24C	0.5348	0.1027	0.0574	0.045*	
C25	0.1098 (4)	0.08582 (15)	0.02536 (17)	0.0353 (5)	
H25A	−0.0395	0.0887	0.0425	0.053*	
H25B	0.1649	0.0348	0.0408	0.053*	
H25C	0.1235	0.0954	−0.0433	0.053*	
C26	0.3406 (3)	0.14549 (12)	0.50876 (14)	0.0245 (4)	
H26A	0.4467	0.1271	0.4640	0.037*	
H26B	0.2341	0.1056	0.5182	0.037*	
H26C	0.4093	0.1578	0.5702	0.037*	
C27	−0.1913 (3)	0.44964 (11)	0.22138 (14)	0.0222 (4)	
C28	−0.3061 (3)	0.42365 (12)	0.13087 (14)	0.0239 (4)	
C29	−0.5102 (4)	0.45424 (13)	0.11046 (16)	0.0297 (5)	
H29A	−0.5735	0.4887	0.1539	0.036*	
C30	−0.6192 (4)	0.43302 (14)	0.02479 (17)	0.0358 (5)	
H30A	−0.7562	0.4530	0.0116	0.043*	
C31	−0.5257 (4)	0.38254 (15)	−0.04068 (17)	0.0389 (6)	
H31A	−0.5993	0.3686	−0.0978	0.047*	
C32	−0.3208 (4)	0.35257 (14)	−0.02083 (16)	0.0345 (5)	
H32A	−0.2572	0.3186	−0.0649	0.041*	
C33	−0.2112 (3)	0.37321 (13)	0.06458 (14)	0.0277 (4)	
H33A	−0.0740	0.3533	0.0775	0.033*	

### Atomic displacement parameters (Å^2^)

**Table:**

	*U*^11^	*U*^22^	*U*^33^	*U*^12^	*U*^13^	*U*^23^
O1	0.0378 (9)	0.0196 (7)	0.0313 (8)	0.0031 (6)	−0.0018 (6)	0.0021 (6)
O2	0.0342 (8)	0.0176 (7)	0.0269 (7)	−0.0002 (6)	0.0027 (6)	−0.0030 (5)
O3	0.0167 (6)	0.0210 (6)	0.0224 (6)	−0.0012 (5)	0.0026 (5)	−0.0013 (5)
O4	0.0187 (7)	0.0196 (6)	0.0243 (6)	−0.0022 (5)	0.0074 (5)	−0.0035 (5)
O5	0.0166 (6)	0.0207 (7)	0.0223 (6)	0.0002 (5)	0.0019 (5)	−0.0005 (5)
C1	0.0195 (9)	0.0175 (9)	0.0204 (8)	−0.0018 (7)	0.0036 (7)	−0.0037 (7)
C2	0.0202 (9)	0.0171 (9)	0.0201 (8)	−0.0001 (7)	0.0051 (6)	−0.0024 (7)
C3	0.0223 (10)	0.0205 (9)	0.0210 (9)	−0.0002 (7)	0.0017 (7)	−0.0001 (7)
C4	0.0201 (10)	0.0197 (9)	0.0218 (9)	−0.0013 (7)	0.0016 (7)	0.0000 (7)
C5	0.0205 (10)	0.0164 (9)	0.0209 (8)	−0.0004 (7)	0.0019 (7)	−0.0020 (7)
C6	0.0172 (9)	0.0184 (9)	0.0198 (8)	−0.0014 (7)	0.0038 (6)	−0.0007 (7)
C7	0.0199 (10)	0.0234 (9)	0.0145 (8)	−0.0001 (7)	0.0053 (7)	0.0013 (7)
C8	0.0174 (9)	0.0232 (9)	0.0228 (9)	−0.0007 (7)	0.0060 (7)	0.0013 (7)
C9	0.0186 (9)	0.0210 (9)	0.0244 (9)	−0.0015 (7)	0.0092 (7)	0.0007 (8)
C10	0.0181 (10)	0.0190 (9)	0.0196 (8)	−0.0005 (7)	0.0031 (7)	−0.0011 (7)
C11	0.0214 (10)	0.0221 (9)	0.0252 (9)	−0.0027 (8)	0.0019 (7)	−0.0052 (8)
C12	0.0235 (10)	0.0188 (10)	0.0284 (10)	0.0026 (7)	0.0032 (8)	−0.0027 (8)
C13	0.0316 (11)	0.0196 (10)	0.0243 (10)	0.0016 (8)	0.0016 (8)	0.0009 (8)
C14	0.0441 (14)	0.0328 (12)	0.0251 (10)	0.0078 (10)	−0.0014 (9)	−0.0042 (9)
C15	0.0347 (12)	0.0320 (12)	0.0292 (11)	0.0016 (9)	−0.0029 (8)	−0.0030 (9)
C16	0.0297 (11)	0.0235 (10)	0.0255 (10)	0.0005 (8)	0.0059 (8)	0.0035 (8)
C17	0.0407 (13)	0.0322 (12)	0.0339 (11)	−0.0022 (10)	0.0106 (9)	−0.0004 (9)
C18A	0.031 (2)	0.025 (2)	0.0165 (18)	−0.0018 (15)	0.0026 (18)	−0.0019 (11)
C19A	0.0319 (18)	0.038 (3)	0.0267 (16)	−0.0004 (15)	0.0072 (11)	−0.0064 (16)
C20A	0.087 (4)	0.037 (2)	0.041 (2)	0.011 (2)	0.033 (2)	0.0124 (16)
C21	0.0172 (9)	0.0237 (9)	0.0210 (9)	−0.0006 (7)	0.0048 (7)	−0.0036 (7)
C22	0.0203 (10)	0.0227 (9)	0.0240 (9)	−0.0014 (7)	0.0041 (7)	−0.0022 (8)
C23	0.0240 (10)	0.0250 (10)	0.0201 (9)	0.0006 (8)	0.0005 (7)	0.0000 (8)
C24	0.0273 (11)	0.0349 (12)	0.0277 (10)	0.0016 (9)	0.0058 (8)	−0.0083 (9)
C25	0.0318 (12)	0.0401 (13)	0.0342 (11)	−0.0028 (10)	0.0039 (9)	−0.0155 (10)
C26	0.0242 (10)	0.0239 (10)	0.0255 (9)	0.0009 (8)	0.0017 (7)	0.0006 (8)
C27	0.0227 (10)	0.0204 (10)	0.0238 (9)	−0.0027 (7)	0.0080 (7)	0.0024 (7)
C28	0.0262 (10)	0.0213 (10)	0.0243 (9)	−0.0018 (8)	0.0029 (7)	0.0065 (8)
C29	0.0303 (11)	0.0275 (11)	0.0313 (11)	0.0010 (8)	0.0029 (8)	0.0084 (8)
C30	0.0304 (12)	0.0370 (12)	0.0396 (12)	−0.0026 (10)	−0.0070 (9)	0.0169 (10)
C31	0.0451 (14)	0.0426 (14)	0.0286 (10)	−0.0095 (10)	−0.0082 (9)	0.0071 (10)
C32	0.0458 (14)	0.0330 (12)	0.0248 (10)	−0.0021 (10)	0.0010 (9)	0.0008 (9)
C33	0.0300 (11)	0.0273 (10)	0.0258 (9)	−0.0016 (8)	0.0034 (8)	0.0035 (8)

### Geometric parameters (Å, º)

**Table:**

O1—C27	1.298 (2)	C17—C18A	1.497 (4)
O1—H1O1	0.8200	C17—H17A	0.9602
O2—C9	1.262 (2)	C17—H17B	0.9603
O3—C7	1.223 (2)	C17—H17C	0.9603
O4—C10	1.389 (2)	C18A—C19A	1.336 (6)
O4—H1O4	0.8200	C18A—C20A	1.488 (6)
O5—C10	1.417 (2)	C19A—H19A	0.9300
O5—C4	1.454 (2)	C19A—H19B	0.9300
C1—C9	1.511 (3)	C20A—H20A	0.9600
C1—C10	1.546 (2)	C20A—H20B	0.9600
C1—C11	1.549 (2)	C20A—H20C	0.9600
C1—C2	1.555 (2)	C18B—C19B	1.324 (12)
C2—C3	1.536 (3)	C18B—C20B	1.479 (12)
C2—H2A	0.9700	C19B—H19C	0.9300
C2—H2B	0.9700	C19B—H19D	0.9300
C3—C16	1.537 (3)	C20B—H20D	0.9600
C3—C4	1.546 (3)	C20B—H20E	0.9600
C3—H3A	0.9800	C20B—H20F	0.9600
C4—C26	1.517 (3)	C21—C22	1.515 (3)
C4—C5	1.544 (2)	C21—H21A	0.9700
C5—C6	1.551 (3)	C21—H21B	0.9700
C5—H5A	0.9700	C22—C23	1.327 (3)
C5—H5B	0.9700	C22—H22A	0.9300
C6—C7	1.519 (3)	C23—C24	1.499 (3)
C6—C21	1.552 (2)	C23—C25	1.505 (3)
C6—C10	1.566 (2)	C24—H24A	0.9600
C7—C8	1.473 (3)	C24—H24B	0.9600
C8—C27	1.402 (3)	C24—H24C	0.9600
C8—C9	1.436 (3)	C25—H25A	0.9600
C11—C12	1.510 (3)	C25—H25B	0.9600
C11—H11A	0.9700	C25—H25C	0.9600
C11—H11B	0.9700	C26—H26A	0.9600
C12—C13	1.333 (3)	C26—H26B	0.9600
C12—H12A	0.9300	C26—H26C	0.9600
C13—C14	1.496 (3)	C27—C28	1.487 (3)
C13—C15	1.506 (3)	C28—C33	1.391 (3)
C14—H14A	0.9600	C28—C29	1.393 (3)
C14—H14B	0.9600	C29—C30	1.392 (3)
C14—H14C	0.9600	C29—H29A	0.9300
C15—H15A	0.9600	C30—C31	1.380 (4)
C15—H15B	0.9600	C30—H30A	0.9300
C15—H15C	0.9600	C31—C32	1.392 (4)
C16—C17	1.541 (3)	C31—H31A	0.9300
C16—H16A	0.9700	C32—C33	1.388 (3)
C16—H16B	0.9700	C32—H32A	0.9300
C17—C18B	1.483 (11)	C33—H33A	0.9300
			
C27—O1—H1O1	109.5	H16A—C16—H16B	107.9
C10—O4—H1O4	109.5	C18B—C17—C16	115.6 (12)
C10—O5—C4	105.31 (13)	C18A—C17—C16	114.9 (3)
C9—C1—C10	107.31 (14)	C18B—C17—H17A	108.5
C9—C1—C11	111.77 (16)	C18A—C17—H17A	117.7
C10—C1—C11	109.10 (15)	C16—C17—H17A	108.3
C9—C1—C2	107.87 (15)	C18B—C17—H17B	116.8
C10—C1—C2	107.82 (14)	C18A—C17—H17B	108.4
C11—C1—C2	112.76 (15)	C16—C17—H17B	108.6
C3—C2—C1	112.52 (15)	H17A—C17—H17B	97.1
C3—C2—H2A	109.1	C18B—C17—H17C	108.9
C1—C2—H2A	109.1	C18A—C17—H17C	99.7
C3—C2—H2B	109.1	C16—C17—H17C	108.0
C1—C2—H2B	109.1	H17A—C17—H17C	107.2
H2A—C2—H2B	107.8	C19A—C18A—C20A	121.0 (3)
C2—C3—C16	110.48 (16)	C19A—C18A—C17	120.8 (4)
C2—C3—C4	109.89 (15)	C20A—C18A—C17	118.2 (4)
C16—C3—C4	114.47 (16)	C18A—C19A—H19A	120.0
C2—C3—H3A	107.2	C18A—C19A—H19B	120.0
C16—C3—H3A	107.2	H19A—C19A—H19B	120.0
C4—C3—H3A	107.2	C19B—C18B—C20B	120.4 (12)
O5—C4—C26	107.49 (15)	C19B—C18B—C17	126.3 (12)
O5—C4—C5	102.80 (14)	C20B—C18B—C17	113.1 (10)
C26—C4—C5	112.76 (16)	C18B—C19B—H19C	120.0
O5—C4—C3	105.83 (14)	C18B—C19B—H19D	120.0
C26—C4—C3	113.40 (15)	H19C—C19B—H19D	120.0
C5—C4—C3	113.54 (15)	C18B—C20B—H20D	109.5
C4—C5—C6	104.59 (14)	C18B—C20B—H20E	109.5
C4—C5—H5A	110.8	H20D—C20B—H20E	109.5
C6—C5—H5A	110.8	C18B—C20B—H20F	109.5
C4—C5—H5B	110.8	H20D—C20B—H20F	109.5
C6—C5—H5B	110.8	H20E—C20B—H20F	109.5
H5A—C5—H5B	108.9	C22—C21—C6	114.87 (15)
C7—C6—C5	112.93 (15)	C22—C21—H21A	108.6
C7—C6—C21	106.98 (14)	C6—C21—H21A	108.6
C5—C6—C21	111.73 (16)	C22—C21—H21B	108.6
C7—C6—C10	112.97 (15)	C6—C21—H21B	108.6
C5—C6—C10	102.64 (14)	H21A—C21—H21B	107.5
C21—C6—C10	109.64 (14)	C23—C22—C21	124.69 (18)
O3—C7—C8	122.18 (17)	C23—C22—H22A	117.7
O3—C7—C6	120.41 (17)	C21—C22—H22A	117.7
C8—C7—C6	117.41 (16)	C22—C23—C24	124.00 (18)
C27—C8—C9	118.67 (18)	C22—C23—C25	121.25 (19)
C27—C8—C7	123.35 (17)	C24—C23—C25	114.76 (17)
C9—C8—C7	116.96 (17)	C23—C24—H24A	109.5
O2—C9—C8	120.92 (18)	C23—C24—H24B	109.5
O2—C9—C1	119.83 (17)	H24A—C24—H24B	109.5
C8—C9—C1	119.16 (17)	C23—C24—H24C	109.5
O4—C10—O5	109.37 (15)	H24A—C24—H24C	109.5
O4—C10—C1	106.11 (15)	H24B—C24—H24C	109.5
O5—C10—C1	109.97 (14)	C23—C25—H25A	109.5
O4—C10—C6	115.32 (14)	C23—C25—H25B	109.5
O5—C10—C6	103.67 (14)	H25A—C25—H25B	109.5
C1—C10—C6	112.36 (14)	C23—C25—H25C	109.5
C12—C11—C1	115.64 (16)	H25A—C25—H25C	109.5
C12—C11—H11A	108.4	H25B—C25—H25C	109.5
C1—C11—H11A	108.4	C4—C26—H26A	109.5
C12—C11—H11B	108.4	C4—C26—H26B	109.5
C1—C11—H11B	108.4	H26A—C26—H26B	109.5
H11A—C11—H11B	107.4	C4—C26—H26C	109.5
C13—C12—C11	126.22 (18)	H26A—C26—H26C	109.5
C13—C12—H12A	116.9	H26B—C26—H26C	109.5
C11—C12—H12A	116.9	O1—C27—C8	120.55 (18)
C12—C13—C14	121.73 (19)	O1—C27—C28	114.10 (17)
C12—C13—C15	123.94 (19)	C8—C27—C28	125.31 (18)
C14—C13—C15	114.29 (18)	C33—C28—C29	119.70 (19)
C13—C14—H14A	109.5	C33—C28—C27	121.89 (19)
C13—C14—H14B	109.5	C29—C28—C27	118.34 (18)
H14A—C14—H14B	109.5	C30—C29—C28	119.8 (2)
C13—C14—H14C	109.5	C30—C29—H29A	120.1
H14A—C14—H14C	109.5	C28—C29—H29A	120.1
H14B—C14—H14C	109.5	C31—C30—C29	120.5 (2)
C13—C15—H15A	109.5	C31—C30—H30A	119.7
C13—C15—H15B	109.5	C29—C30—H30A	119.7
H15A—C15—H15B	109.5	C30—C31—C32	119.7 (2)
C13—C15—H15C	109.5	C30—C31—H31A	120.1
H15A—C15—H15C	109.5	C32—C31—H31A	120.1
H15B—C15—H15C	109.5	C33—C32—C31	120.2 (2)
C3—C16—C17	111.89 (17)	C33—C32—H32A	119.9
C3—C16—H16A	109.2	C31—C32—H32A	119.9
C17—C16—H16A	109.2	C32—C33—C28	120.1 (2)
C3—C16—H16B	109.2	C32—C33—H33A	120.0
C17—C16—H16B	109.2	C28—C33—H33A	119.9
			
C9—C1—C2—C3	160.42 (15)	C2—C1—C10—C6	56.49 (19)
C10—C1—C2—C3	44.83 (19)	C7—C6—C10—O4	−91.58 (18)
C11—C1—C2—C3	−75.68 (19)	C5—C6—C10—O4	146.50 (15)
C1—C2—C3—C16	−175.28 (15)	C21—C6—C10—O4	27.6 (2)
C1—C2—C3—C4	−48.0 (2)	C7—C6—C10—O5	148.89 (14)
C10—O5—C4—C26	163.91 (14)	C5—C6—C10—O5	26.98 (17)
C10—O5—C4—C5	44.74 (17)	C21—C6—C10—O5	−91.90 (17)
C10—O5—C4—C3	−74.61 (16)	C7—C6—C10—C1	30.2 (2)
C2—C3—C4—O5	61.30 (18)	C5—C6—C10—C1	−91.72 (17)
C16—C3—C4—O5	−173.72 (15)	C21—C6—C10—C1	149.40 (16)
C2—C3—C4—C26	178.88 (16)	C9—C1—C11—C12	78.7 (2)
C16—C3—C4—C26	−56.1 (2)	C10—C1—C11—C12	−162.80 (16)
C2—C3—C4—C5	−50.7 (2)	C2—C1—C11—C12	−43.0 (2)
C16—C3—C4—C5	74.3 (2)	C1—C11—C12—C13	153.3 (2)
O5—C4—C5—C6	−25.42 (17)	C11—C12—C13—C14	179.2 (2)
C26—C4—C5—C6	−140.85 (15)	C11—C12—C13—C15	2.0 (3)
C3—C4—C5—C6	88.42 (18)	C2—C3—C16—C17	−66.0 (2)
C4—C5—C6—C7	−122.38 (16)	C4—C3—C16—C17	169.32 (16)
C4—C5—C6—C21	116.96 (16)	C3—C16—C17—C18B	−168.3 (8)
C4—C5—C6—C10	−0.44 (18)	C3—C16—C17—C18A	179.6 (3)
C5—C6—C7—O3	−44.1 (2)	C18B—C17—C18A—C19A	137 (8)
C21—C6—C7—O3	79.2 (2)	C16—C17—C18A—C19A	−126.4 (5)
C10—C6—C7—O3	−160.05 (15)	C18B—C17—C18A—C20A	−43 (7)
C5—C6—C7—C8	135.35 (16)	C16—C17—C18A—C20A	53.4 (6)
C21—C6—C7—C8	−101.31 (18)	C18A—C17—C18B—C19B	−21 (5)
C10—C6—C7—C8	19.4 (2)	C16—C17—C18B—C19B	−110 (3)
O3—C7—C8—C27	−28.6 (3)	C18A—C17—C18B—C20B	163 (9)
C6—C7—C8—C27	151.94 (17)	C16—C17—C18B—C20B	74 (2)
O3—C7—C8—C9	139.71 (18)	C7—C6—C21—C22	−67.3 (2)
C6—C7—C8—C9	−39.8 (2)	C5—C6—C21—C22	56.8 (2)
C27—C8—C9—O2	−1.0 (3)	C10—C6—C21—C22	169.87 (15)
C7—C8—C9—O2	−169.84 (17)	C6—C21—C22—C23	−161.87 (19)
C27—C8—C9—C1	175.66 (16)	C21—C22—C23—C24	−0.6 (3)
C7—C8—C9—C1	6.8 (2)	C21—C22—C23—C25	178.91 (19)
C10—C1—C9—O2	−141.69 (17)	C9—C8—C27—O1	−0.3 (3)
C11—C1—C9—O2	−22.1 (2)	C7—C8—C27—O1	167.77 (18)
C2—C1—C9—O2	102.38 (19)	C9—C8—C27—C28	177.48 (17)
C10—C1—C9—C8	41.6 (2)	C7—C8—C27—C28	−14.4 (3)
C11—C1—C9—C8	161.21 (16)	O1—C27—C28—C33	137.03 (19)
C2—C1—C9—C8	−74.29 (19)	C8—C27—C28—C33	−40.9 (3)
C4—O5—C10—O4	−168.82 (14)	O1—C27—C28—C29	−39.7 (3)
C4—O5—C10—C1	75.03 (16)	C8—C27—C28—C29	142.3 (2)
C4—O5—C10—C6	−45.31 (16)	C33—C28—C29—C30	1.2 (3)
C9—C1—C10—O4	67.41 (17)	C27—C28—C29—C30	177.98 (19)
C11—C1—C10—O4	−53.85 (18)	C28—C29—C30—C31	−0.7 (3)
C2—C1—C10—O4	−176.63 (14)	C29—C30—C31—C32	0.1 (3)
C9—C1—C10—O5	−174.40 (15)	C30—C31—C32—C33	0.1 (4)
C11—C1—C10—O5	64.34 (18)	C31—C32—C33—C28	0.3 (3)
C2—C1—C10—O5	−58.44 (18)	C29—C28—C33—C32	−1.0 (3)
C9—C1—C10—C6	−59.47 (19)	C27—C28—C33—C32	−177.67 (19)
C11—C1—C10—C6	179.27 (15)		

### Hydrogen-bond geometry (Å, º)

*Cg*4 is the centroid of the C28–C33 ring.

**Table:**

*D*—H···*A*	*D*—H	H···*A*	*D*···*A*	*D*—H···*A*
O1—H1*O*1···O2	0.82	1.70	2.4446 (19)	151
O4—H1*O*4···O3^i^	0.82	1.97	2.7499 (18)	159
C21—H21*B*···O3^i^	0.97	2.48	3.283 (2)	139
C25—H25*B*···*Cg*4^ii^	0.96	2.99	3.786 (3)	142

Symmetry codes: (i) *x*+1, *y*, *z*; (ii) −*x*, *y*−1/2, −*z*.
